# The Concours—Clinical Chair of Surgery, Paris

**Published:** 1835-01-01

**Authors:** 


### THE CONCOURS—CLINICAL CHAIR OF SURGERY, PARIS.

1. Parallele entre la Taille, et la Lithotritie. Par *P. F. Blandin.* Octavo, pp. 167. Paris, 1834.

2. Des Hemorroides, et de la Chute du Rectum. Par *A. Lepelletier de la Sarthe.* Octavo, pp. 168. Paris, 1834.

3. Des Diverses Methodes, et des differens Procédés pour l'Obliteration des Arteres, &c. Par *I. Lisfranc.* Octavo, pp. 154. Paris, 1834.

The above are the titles of three of the Memoirs presented by the Candidates for the Chair of Clinical Surgery, at the late Concours held in Paris; the other two have not yet been sent to us, and we regret this the more, as that of M. Velpeau, the successful candidate, is one of these.

M. M. Blandin and Lisfranc are hospital surgeons in Paris; the former is attached to the Hôpital Beaujon, and is favorably known to the profession by his treatise on abdominal effusions, and by his larger work on topographical anatomy; the latter is the surgeon-in-chief to the Hôpital de la Pitié, and for many years has enjoyed perhaps the most extensive reputation of any teacher in Europe, as an able lecturer on surgical anatomy. The subjects of the memoirs are of great practical interest, and as it cannot fail to be instructive to the medical public to be made acquainted with the sentiments of those who are justly celebrated for their talents and research, we intend devoting a few pages to the examination of two at least of these memoirs, the first and the third; the second, namely, that of M. Lepelletier, we regret to be obliged to declare is of very inferior merit.

### 1. On Lithotomy and Lithotrity.

It is really quite amusing to read M. Blandin's descant on the "grandes difficultés" which environ his labours on every hand. The subject which has been imposed upon him is, we are told, "immense," and "d'une étendue effrayante," and would require "d'enormes folios" to do it justice. How then is it to be expected that even he can, in the course of nine days, (the term, it seems, allotted by the Concours for the "getting up" of the essays of the candidates,) achieve a work which demands many months, if not years, for its right execution? With the assistance of "ces flambeaux si precieux," synthesis and analysis, we are led through the gloomy mazes of the vast profound, under the guiding direction of our learned author; but in order to reach this happy end, we must first submit to wade through upwards of thirty pages of a "developpement historique" of the operations of lithotomy and of lithotrity. What the history of the operations has to do with the comparison of their respective merits we must not, we suppose, presume to inquire. At the very threshold of our journey we encounter these words: "there are few branches of human knowledge which need more lengthened and varied labours than does lithotomy," an announcement almost as formidable to the poor reader as the inscription "per me si va per citta dolente" over the portal of the Inferno was to Dante; for "this operation, the ne plus ultra of surgical dexterity, from the time of the ancient Egyptians down almost to the present day, has baffled the ablest efforts of men of all countries to bring to a state of comparative perfection." M. Blandin commences, as in duty bound, with the Coan Sage, and tells us that he somewhere in his writings recommends that no one should be permitted to cut for the stone, unless he applied himself exclusively to this *trade.* After Hippocrates, we are introduced to Ammonius, and then to Celsus, who, we are informed, anticipated Baron Heurteloup in his lithotritic inventions.

Our readers would not thank us for telling them of the apparatus, minor and major, and of the various methods in which different authors have practised the "taille lateralisée," or lateral* operation of lithotomy. Even M. Blandin admits that "on n'espere certainement pas que nous fassions ici l'histoire des voyages de Frere Jacques de Besançon à Paris;" for our part, we should answer not. The bilateral operation invented by Celsus had ceased to be heard of, until M. M. Chaussier and Ribes, in 1805, recalled the attention of the profession to its merits. It has subsequently been much improved by Dupuytren, who has contrived a double-bladed lithotome, to divide both sides of the cervix of the bladder at the same time. The object aimed at in the bilateral, as also in the quadrilateral (proposed by M. Vidal de Cassis in 1825) operations is the making of an opening into the bladder for the extraction of large calculi, without extending the incision much beyond the prostate, and without the consequent hazard of the division of the venous plexuses of the neck of the bladder, and of the sheath which surrounds them; for it has been to this accident that some of the most alarming effects of lithotomy, such as the effusion of the urine into the cellular texture, the formation of confined abscesses, and the establishment of fatal phlebitis, have been generally, and we believe correctly, attributed.

At page 24 of the "Parallele," commences the "developpement historique" of lithotrity; and as a matter of course it is prefaced with some French persiflage about the "rapidité toute magique et à peine croyable," with which this operation has been brought to perfection: "jamais invention humaine n'a marché d'un pas si precipité dans la voie de progrès; mais aussi quelle autre invention a eu plus d'importance?" We find that some very distinct allusions are made to lithotrity in some old authors; thus Benedictus, in 1533, uses these words—"aliqui intus sine plagâ lapidem conterunt ferreis instrumentis," and Sanctorius, in 1580, writes—"specillum sagittatum immittit chirurgus et calculum dividit." Paré, Hildanus, and others describe forceps "a trois branches," for extracting calculi from the urethra, and even from the bladder.

---

* It is worthy of notice, that what is called by the French the "taille laterale" is very different from our lateral operation, which corresponds with their "t. lateralisée." In the former, the lateral portion of the cervix, or bas-fond of the bladder is opened at once, without dividing the membranous portion of the bladder, or the prostate gland. M. Blandin condemns it as the most imperfect of all the operations of lithotomy.

According to Blandin's account, the merit of having brought lithotrity into notice of late years belongs to M. Gruithuisen, a Bavarian physician, who was engaged in endeavouring to realise the proposal of M. M. Vauquelin and Fourcroy to dissolve urinary calculi by introducing menstrua into the bladder. He had contrived an instrument to seize the stone, and to bore it in several places, with the view of subjecting it more effectually to the action of the solvent. In 1819, M. Eldgerton contrived an instrument which opened into two parts or blades, with which the calculus was to be seized, and then it was subjected to the action of a file. Our author shews his good taste in not venturing to decide on the comparative claims of his cotemporaries, Civiale, Amussat, and Leroy d'Etiolles, to the priority of certain inventions, and he merely says that ever since the second of these surgeons proved more distinctly than had been done before, that the bladder may be reached with great ease by straight sounds, the great impulse to all the subsequent improvements of lithotrity may be traced.

By a singular coincidence, M. Amussat and M. Leroy presented to the Academy on the same day, viz. 13th June, 1822, instruments which they had contrived for perforating and crushing the stone in the bladder. Since that date Baron Heurteloup has done much to perfect the instrument proposed by Amussat, and he has also invented some of his own. This ingenious surgeon has the high merit of having introduced the latest, and perhaps the most valuable improvement of the operation: we mean that of crushing the stone by percussion, just in the same way as we should break a pebble with a hammer. Did our limits permit us, we should have alluded to the labours of several other zealous surgeons; but we must hurry on to the third chapter, which will not however detain us long; it is headed, "Parallele Historique entre la Taille et la Lithotritie!!" And now we have a second edition of some common and schoolboy-like remarks on the snail-pace of the progress of the former, and on the giant strides of the latter from its birth to the maturity of its manhood. What with the "progrès immense," the "ingenieuse activité du monde chirurgical," and something about the "cendres des inventeurs de la cystotomie," and the "berçeau de l'humanité," we are quite bedazzled with the brilliancy of M. Blandin's rhetoric, and are apt to forget its subject-matter. Quite Frenchman-like, he must have a passing allusion at some prurient details; and we are therefore gravely informed that among the causes which retarded during many centuries the improvement of lithotomy, may be mentioned that prejudice which the chaste Abulkasem alludes to in these words:—"quand une femme avait la pierre, il faillait appeller une matrone, parcequ'aucun homme n'avait le droit de porter les yeux sur les organes genitaux du sexe." Our author adds, "heureusement pour les femmes aujourdhui ce ne sont pas des matrones qui les debarassent de leurs calculs."

The next chapter is occupied with a comparison of the two operations in regard to the pain suffered by the patient at and after their performance, and M. B. comes to the conclusion that, on the whole, lithotrity is in most cases attended with more pain than the cutting with the knife. The introduction of the large sound along the urethra is often very difficult, causing most severe pain, especially at the entrance of the canal, (which it is occasionally necessary to enlarge with the knife,) and at the neck of the bladder, and a copious hæmorrhage has in more instances than one been the result; then, again, the manœuvre of seizing the calculus or its fragments, and the subsequent operation of crushing them is, even in the most practised hands, not devoid of suffering: to these sources of distress we must add the longer time necessary for the performance of the operation, and the repetition of the séances necessary for its completion. It is, however, but candid to admit that the pain of introducing the full-sized sound may be much diminished by dilating the canal gradually for several days or weeks previous to the operation, and that the subsequent manœuvres are generally performed with admirable dexterity by that prince of lithotritists, the Baron Heurteloup. What Hippocrates said of lithotomy ought to be transferred to lithotrity, viz. that it is an operation to be attempted only by those who devote themselves exclusively to its performance; and certainly, from what we have witnessed, we are decidedly of opinion that it can never be made an operation which the general surgeon will be able to undertake with as much confidence as he does lithotomy. This, however, ought not to be regarded as any substantial objection to its introduction, as a substitute for the use of cutting instruments in numerous cases of urinary calculus. M. Blandin is of opinion that the very consequences of the incisions of lithotomy are often useful to the final recovery of the patient;—the long gorged and irritated vessels of the bladder are relieved by the hæmorrhage; and hence catarrhs and even induration of its mucous surface are not unfrequently got rid of, after the recovery of the patient from the immediate effects of the operation; whereas, after a successful lithotrity, a sense of uneasiness and a tenderness in the hypogastric region, enuresis, and pain in voiding the water, which may continue to be at the same time glairy, or even bloody for a considerable time, are common sequences. It may, however, be replied to this objection, and we admit that the answer is satisfactory, that if lithotrity be performed in the early period of the disease, when the calculus is small and the bladder quite healthy, no such disagreeable results are ever known to occur.

The minute details which M. Blandin has thought proper to enumerate, respecting the various accidents which attend and follow the operation of lithotomy, are misplaced in a "parallele" or comparative review of the merits of the two operations. It is not necessary for us to state that a troublesome and even alarming hæmorrhage may be caused by the division of the veins of the bulb, and of the cervix of the bladder, or of the transverse artery of the perineum, or of the internal pudic itself, of which a memorable example occurred in the practice of M. Roux in 1822, when the operator succeeded in arresting the hæmorrhage by tying the trunk in its course along the inner edge of the tuberosity of the ischium.* We are pleased to

---

* A few remarks on secondary or consecutive hæmorrhage after lithotomy are deserving of notice, as their correctness is warranted by the authority of M. Cruveilhier. According to this very eminent pathologist, one of the most frequent causes of this species of hæmorrhage is inflammation of, and the consequent suppuration within the divided bloodvessels. The coagulum becomes detached from the internal surface, and there is then no obstacle to the discharge of the fluid blood. In such a state of the parts, the application of a ligature may probably be of no avail, as the parietes of the vessels are soft and easily lacerable; and even cauterization, and the plug, although preferable to the ligature, will in many cases be equally inefficacious.

observe that our intelligent author disapproves of making the internal incision extend beyond the limits of the prostate; his rule is "aller jusque vers les limites de la prostate, mais ne les depasser jamais." The most skilful lithotomists in this country appreciate the justness of the observation; and our readers may be benefited by re-perusing the valuable remarks of Sir B. Brodie on this subject, in one of our late numbers. The other accidents, such as wounding of the rectum, of the peritoneum, (especially in the high and in the recto-vesical operations,) of one of the vesiculæ seminales, or a vas deferens, of the extremity of the ureter, and, above all, of the superior perineal aponeurosis, (fascia pelvia of Cloquet,) are of occasional occurrence. We have pointed particularly to the division of the perineal fascia, because it is by avoiding this sheath, that we steer clear of most of the other evils, as well as of that very fatal one, the infiltration of the urine into the surrounding textures. The importance, therefore, of not prolonging the incision of the neck of the bladder beyond the limits of the prostate gland, (which is invested outwardly with this fibrous lamina,) cannot be too strongly urged. It may be said that when the calculus is large, the wound is apt to be lacerated during its extraction; and so it certainly may be sometimes, if no modification of the common lateral operation, such as nicking the edges of the vesical incision, or adopting the bilateral method proposed by Dupuytren, be had recourse to; but the scientific surgeon will shew this superiority over the mere "handworkman," by accommodating the circumstances of the operation to the peculiarities of each case.

Certain it is, that one of the most frequent causes of death after lithotomy is the infiltration of urine into the cellular sub-peritoneal tissue around the neck of the bladder and in the perineum; and it is equally certain that this very fatal accident may generally be traced to a too free incision of the neck of the bladder, or to the internal and external incisions not being made in the same line and direction. The precept of M. Lisfranc is "à inciser les parties molles perineales, depuis le col de la vessie jusqu'à la peau, dans une etendue au moins egale, superieure même, s'il est possible, a l'ouverture que procure le debridement du col de la vessie."

There is a consecutive accident, which is of more frequent occurrence, in M. Blandin's opinion, than has been hitherto supposed; namely, inflammation of the veins at the neck of the bladder. He has detected it in six cases, since his attention was first directed to the inquiry. These veins and also those of the perineum are numerous, some of them large, having a semi-erectile texture, and so lodged in and confined by the perineal aponeurosis, that when divided, they remain with open mouths, and are therefore more apt to be exposed to the irritation of the air from without, and of the urine as it flows out of the wound.

One species, or, perhaps more correctly we should say, specimen of perineal phlebitis, is the inflammation of the erectile tissue of the urethra; for all erectile tissues are essentially venous in their formation. When the bulb becomes inflamed, the venæ dorsales penis, and also those of the prostatic plexus, very frequently become similarly affected, indicating thus the character of the original mischief.

Our limits permit us only, en passant, to allude to fistulæ in the perineum as one of the occasional evils attendant on lithotomy, and equally brief must be our notice of the ischuria, and more frequently the incontinence of the urine which sometimes follows. We must now proceed to enumerate some of the more frequent accidents which may be induced by the operation of lithotrity; and we shall probably be tempted to describe these at greater length, as the results of this operation are far less generally known to the profession than the dangers of lithotomy. M. Blandin enumerates no fewer than thirteen, viz. alarming nervous symptoms, inflammation of the urinary passages, of the prostate, of the vesiculæ seminales, of the peritoneum, of the veins of the pelvis, injury of the mucous coat of the bladder and hæmaturia, perforation of the bladder, infiltration of urine, retention and incontinence of urine, urinary fistulæ, the breaking of some of the instruments within the bladder, and, lastly, the reproduction of the calculus. Not one member of this catalogue, however, can be charged as peculiar to lithotrity; but yet it is well to know, that all of them have been known to occur. We shall subjoin a few illustrative remarks, but have not room to insert the cases reported in our author's treatise. The sensibility of the urethra and bladder is in some calculous patients so extreme, and the agony produced by the introduction of large-sized instruments so great, that very serious accidents may be produced if we persevere in our attempts. A case, which occurred in the practice of Leroy d'Etiolles, is alluded to. The man died on the 4th day, after an attempt had been made to seize the calculus with the "brise-pierre" of M. Heurteloup. And in Larrey's report on the account of the calculous patients treated by M. Civiale at the Hôpital Necker, we observe that one of them was seized with alarming nervous symptoms after the first séance had been undergone, and died on the third day afterwards.

A much more common source of danger is the inflammation of the urinary passages; this indeed is the capital objection to lithotrity; as it can scarcely ever be performed, even in favorable cases, without being followed by a certain degree of inflammatory irritation of the urethra and neck of the bladder: hence the frequent desire and painful efforts to make water, and the mucous turbidity of this secretion, after each séance. Whenever this irritation exceeds a certain degree, the safety of the patient demands that the operation be not repeated for some time. In some cases, in addition to the local symptoms, we find that the constitution sympathises so much, that a sort of nervous fever, attended with extreme depression of the vital powers, comes on; and in other cases, the inflammation has been known to extend to the ureters and kidneys, an event of very alarming danger.

Phlebitis of the neck of the bladder is not of such frequent occurrence after lithotrity as it is after lithotomy. The following however is an example which happened in M. Blandin's own practice. A middle-aged man had, in a foolish moment, introduced into his urethra a stalk of long grass, the end of which broke off in his bladder, and gave rise to the formation of a calculus. He went to the Hôtel Dieu, and M. M. Dupuytren and Breschet regarding the case as one favorable for the operation of crushing the stone, remitted him to the care of M. Blandin. The sound detected the presence of several calculi: two were readily crushed by means of the forceps and drill; a third, being much harder, required the use of the bow. While M. B. was using this, the patient on a sudden made a violent struggle, and shrunk back so much from the instrument, that its extremity was drawn within the urethra; fortunately, the calculus had by this time been crushed, and the blades were closed, else the neck of the bladder must inevitably have been lacerated. M. Blandin candidly confesses that he had omitted to secure the body of the patient to the bed with the shoulder-strap. Two other séances were successively undergone, and several calculi crushed and discharged; but unluckily a fragment of one larger than the rest became impacted in the membranous portion of the urethra, and could not be dislodged; and, although it did not much obstruct the passage of the urine, it prevented the introduction of any instrument into the bladder. Things continued in this state for several days, when the patient, upon leaving a warm-bath, caught cold, and was seized with a violent pneumonia, which speedily proved fatal. On dissection, the calculus was found in the membranous portion of the urethra, which, as well as the prostatic portion, had become much inflamed; the adjacent veins were found to contain pus; numerous small abscesses were observed disseminated through the substance of both lungs;—a score, at least, of small calculi were found in the bladder.

The injury of the mucous coats of the urethra and bladder was certainly of more frequent occurrence, when the forceps " a trois branches" was generally used, than it has been of late. This accident is, as we may guess, most apt to happen when the bladder is much contracted, and resists distention. Not to talk of pieces of the mucous membranes brought away by the extremity of the litholabe. we may assert with confidence that it is sometimes scarcely possible to avoid including the uvula vesicæ between the branches of the forceps, especially as this little appendage is very often enlarged in calculous patients. The improvements introduced by M. M. Jacobson and Heurteloup have however considerably diminished the risk of this accident. M. Tanchore was once witness of a very copious hæmorrhage, which had been produced by laceration of the urethra in attempting lithotrity. The dreadful accident of fairly perforating the walls of the bladder is in the present day, and especially when the instruments for percussion and compression are used, scarcely to be admitted as one of the evils which attend this operation. M. Breschet has related one such melancholy case.

The occasional occurrence of such accidents as urinary infiltration, the formation of urinary fistulæ, and incontinence of urine, need no illustrative remarks; and even the last member in the catalogue of evils, the reproduction of the calculus, may in the present day be almost excluded from consideration.

The following summary is given by M. Blandin of the comparative advantages and disadvantages of the two operations.

1. Hæmorrhage is much more frequent and more alarming after lithotomy than after lithotrity.

2. The rectum, peritoneum, and other important organs may be wounded in lithotomy. These accidents have been known to occur in lithotrity; but not certainly with the present improved instruments.

3. Urinary infiltration is not uncommon after lithotomy;—rare, very rare, after lithotrity.

4. Phlebitis is of infinitely more frequent occurrence after lithotomy than after lithotrity; indeed only one instance after the latter operation is on record.

The same remark is applicable to the accidents of peritonitis and of urinary fistulæ, &c. &c.

The preceding objections are unfavorable to lithotomy; but let us now consider those which may be urged against its rival.

1. The pain and nervous accidents are, on the whole, more severe, more prolonged, and of more frequent occurrence after lithotrity than after lithotomy.

2. Cystitis is a more frequent sequence of lithotrity than of lithotomy.

3. The inflammation of the prostate terminating in abscesses, and producing retention of urine and other serious accidents, is "of greater importance" after lithotrity than after lithotomy.

4. The bladder may be wounded in lithotrity more readily than in lithotomy.

5. Lithotrity may be reproached with the risk of the extremity of an instrument being broken off, and left behind in the bladder.

6. The chance of reproduction of the calculus is greater after lithotrity than after lithotomy; but here it is but fair to state, that the statistic reports of M. Civiale are directly opposed to this conclusion.

We have hitherto not alluded to the conditions which may render the one or the other operation advisable or not, in particular cases, and we shall now, therefore, briefly allude to these. As a general remark, we may state that lithotrity is better adapted to those cases in which there is only one or two, than when there are numerous calculi present in the bladder. M. Civiale, indeed, tells us that, in one of his patients, he crushed and discharged no fewer than forty calculi, and M. Leroy has been successful in three cases, in which 15, 20, and 30 calculi had been present. When the calculus is very large, and especially when it is embraced by the walls of the bladder, lithotrity is scarcely admissible; such cases must be left to the lithotomist.* The difficulty of seizing a large calculus without irritating, or even pinching, the coats of the bladder is not the only objection; the length of the treatment, the numerous séances required, and the consequent repeated irritation of the urinary passages, add much to the distress, and even to the danger of the case. The sentiments of M. Blandin on this subject are clearly expressed in the following sentence. "The important point to determine, in reference to the adoption of lithotomy or of lithotrity, in cases of large calculi is, whether the patient is able to bear the repetition of numerous séances, and the contingent inconveniences and accidents of these, or the operation of at once extracting the calculus by cutting, with least danger to his constitution. In the present state of our knowledge, I do not hesitate to decide the question in favour of lithotomy."

The flattened shape of a calculus has, we are told, in more instances than one, precluded the removal of it by lithotrity; but it is right to state that, in the two cases adduced in illustration by M. Blandin, the bladder was unsound in both patients; and, moreover, that the perforating, and not the percussing, instruments were employed by the operator. Some of the calculi of the oxalate of lime are of such extreme hardness, that they resist all attempts to break them with the most approved lithotritic instruments; our

* In our last Number, indeed, there is a notice of a successful operation by Mr. Costello, of London, in a case in which the calculus was very large, being 3¼ inches in its long, and 2¾ inches in its short axis.—REV.

author has met with one case of this kind, and reports another from the practice of M. Guersent. Having thus briefly alluded to the varying conditions of the calculi themselves, as they may influence the decision of the patient or surgeon, as to the operation to be adopted, our attention is directed next to the conditions of the urinary passages, with the same view, and we shall find, that in not a few cases is lithotrity nearly inapplicable: when the calculus is at all impacted into the opening of the urethra, or encysted—or when there is a strictured state of the urethra—or when the bladder is affected with paralysis (and, therefore, has no power to expel the debris and fragments), or when it is much thickened, and in a catarrhal, or very irritable state—or when the prostate gland or uvula vesicæ is much enlarged, lithotrity is not to be recommended. By far the most important of these morbid conditions to be attended to, is that in which the bladder is extremely irritable, and there is an evident tendency to inflammation, either of it or of the kidneys; under such circumstances, the cutting operation is the safer of the two, as the attendant hæmorrhage not unfrequently relieves that condition of the system on which these accidents generally depend. With respect to the existence of paralysis of the bladder, in calculous cases, it deserves notice, that Professor Roux has met with several cases, in his own practice, where the loss of nervous power returned soon after the stone had been extracted by lithotomy.

The last set of circumstances to be considered, in reference to the two operations, is that which respects the age, sex, and constitution of the patients.

For very obvious reasons, lithotrity is not well applicable in young children, and the rule laid down several years ago, by MM. Dupuytren, Roux, and Amussat is probably near the truth, viz. "jusqu'à douze ou quinze ans environ, les calculs vesicaux doivent etre le plus souvent traités par la lithotomie;" the irritability of the bladder, its small antero-posterior capacity, the narrowness of the urethra, and the difficulty of confining the young patient, are all so many unfavourable circumstances. To shew, however, that lithotrity may be performed with success at very early periods of life, we may direct our readers to a report of some cases, recently presented by M. Segalas to the Academy of Medicine. In advanced life, the urinary bladder, provided it is healthy in its texture, is in the most favourable condition for the operation of lithotrity; it has become more capacious and much less irritable, and less disposed to be affected with inflammation, and, moreover, the urethra has become so much dilated, that the requisite instruments may be introduced with comparative facility. Hence it is, that by far the greater number of cases on record have occurred in elderly patients; thus, out of 38 reported by M. Heurteloup, 23 were in patients from 60 to 80 years of age. In only one of these patients was the operation fatal.

As to the constitution of our patients, the remarks of M. Blandin are extremely pertinent: he gives the preference to lithotrity (barring any important objection) whenever the system is very highly nervous, and the mind is much depressed and alarmed at the idea of being "cut," and he alludes to some melancholy cases, wherein lithotomy had been very speedily followed by death, apparently from the mere shock of "mental perturbation, and without any serious corporeal lesion." It is proper, however, that the surgeon, for the sake of his own character, as well as for the safety of his patient, should always bear in mind the practically-important fact, that the chances of success of lithotomy are considerably impaired, if the operation be performed after previous attempts to remove the stone by lithotrity. Much sagacity, discrimination, and calm resolve are, therefore, requisite, under the circumstances we have been alluding to.

We have left little space to enter upon any tabular comparison of the two operations, drawn from the registers, which hitherto have been made public. At best, this method of arriving at accuracy of conclusion is more specious than trustworthy. It may possibly be thought by some, that if the lithotritist can display a greater number of successful cases, out of a given number operated upon, than the lithotomist, the question of inquiry is at once settled; but not so—the cases may not be similar and equally favorable; the lithotritist generally selects those which he deems to be accommodated to his operation, and rejects the others, which are then entrusted to the ordinary surgeon. Besides this, it has occurred in more than one instance, that lithotrity has been attempted, but has failed, and that the cure of the patient has then been effected ultimately by the cutting operation. These considerations must, therefore, be taken into account, in discussing the mere question of the comparative merits of the two operations; but, after all, they interest rather the speculative disputant than the man engaged in practice.

If we collect the cases of lithotomy reported by F. Cosme, Douglas, Cheselden, Dr. Marcet, and those which stand on the books of the Hôtel Dieu and of the Charité Hospital, from the year 1720 to 1727, an aggregate of 1431 operations, performed in different countries and after different methods (and be it recollected, these methods not so approved as those now practised), is obtained; and of this number, we find that 1085 were successful and 346 fatal, being in the proportion of one death in rather more than three of the cases. Now let us compare this estimate with the results of lithotrity, as far as we can ascertain them from the work of M. Baucal, the report of MM. Larrey and Double, on the Comptes-rendus of the Hôpital Necker by M. Civiale, in the years 1831-2-3, and from the Memoirs of Baron Heurteloup:—These various sources furnish us with a catalogue of 124 cases of lithotrity, performed, indeed, not in the same, but after different methods. Of this number, we find that 86 patients were cured, 30 died, and 8 remained unrelieved; the proportion of deaths is, therefore, one in rather less than every three cases operated on. Hence it appears, that lithotomy has a decided advantage over its rival; and this advantage will be found to be more conspicuous, if we compare the more recent registers of lithotomy cases, such as those reported in the Leçons Cliniques of Dupuytren, and in the works of M. Belmas, of Vacca Berlinghieri, and others, with the above aggregate of lithotrity cases: the proportion of deaths being less than one in four patients; and it is worthy of especial notice, that among the 537 cases drawn from the last-mentioned sources, there were a good many of the high operation, and also several in which lithotrity had been unsuccessfully attempted. As our only motive in giving these details is the wish to arrive at truth, it is a duty which we owe to Baron Heurteloup to state that the results of his experience, since he contrived and adopted the percussing or hammering instruments, have been greatly more successful than those of M. Civiale, as detailed in the report of the Hôpital Necker, already alluded to. The splendid success of effecting cures in 37 out of 38 cases, is a proud testimonial of the skill and dexterity of the Baron, and forms a striking contrast to the 27 cures, out of 43 cases, detailed in M. Civiale's memoir.

Should lithotrity henceforth be as successful, even in picked cases, as it has lately been in Heurteloup's practice, the most sceptical must admit that, under certain circumstances at least, it must bear the palm from its ancient rival. In conclusion, we may confidently aver—

1. That lithotrity is one of the most brilliant achievements of modern surgery—that it is to be viewed at once as a rival and as "la sœur" of lithotomy; but that it can never supersede it altogether.

2. That lithotrity, if resorted to in all cases, to the exclusion of lithotomy, would very probably be more dangerous and unsuccessful than this last operation has been.

3. That the operation of breaking and crushing a urinary calculus is much more easy, expeditious, and fortunate than that of drilling and boring it; that the former of these methods has been more successful in its results than lithotomy has almost ever been; and that the latter has been less successful than it is usually.

4. That the cure effected by removing a calculus by lithotomy is probably more perfect, and ultimately more complete, than that ever obtained by lithotrity.

5. That we cannot determine what might be the success of lithotomy, if the surgeon was permitted to select his cases, as the lithotritist has hitherto done, and no doubt always will do.

---

II. Des Hemorrhoides, et de la Chute du Rectum. Par A. Lepelletier de la Sarthe, pp. 165.

This is certainly the least praiseworthy of all these Concours essays; it is not one degree better than the common run of inaugural theses or dissertations; and, indeed, the author seems not to profess to offer his own opinions, but merely to deliver those of other writers. His treatise is, therefore, nothing but a mere compilation, and as we have this already, in such able works as Cooper's Dictionary, and the Dictionnaire de Médécine et Chirurgie, M. Lepelletier might as well have saved himself the trouble and expense of printing 165 pages. Perhaps he thinks that the want of originality may be compensated for by a display of his literary lore; for there is appended to the "omnium gatherum" of his remarks on piles, and prolapsus of the rectum, a list of nearly 300 works on these subjects, commencing, as usual, from Dr. Hippocrates, in the year 400 B. C. to M. Lepelletier de la Sarthe, in 1834. Such being the character of this Essay, we are reluctantly compelled to pass it by; but, perhaps, may extract a few of the reported cases for our Periscopic department.

---

III. Des diverses Methodes, et des differens Procédés pour l'Obliteration des Arteres, &c. Par I. Lisfranc.

It may be proper to premise a few remarks which M. L. has made on the anatomical structure of the middle coat of the arteries. This coat is composed of spiral fibres, which pass round the tube of the vessel two or three times, and perhaps oftener, although they cannot be easily followed; they are of a yellowish colour and of a dry texture in the aorta and large arteries, and are much redder and moister in the smaller. Some authors have compared this tissue to the muscular, others to the elastic fibrous substance of the vertebræ; perhaps neither description is right. Suffice it for us to say, that the spiral arrangement accounts readily for the property of extensibility which the middle tunic possesses lengthways, and for the power of retraction which it exhibits when the vessel is cut or torn though. It differs certainly from the common fibrous elastic tissue, by the extreme readiness with which it is divided on pressure, whether this pressure be made with a ligature, forceps, or even with the finger-nail. Let us now attend to the internal tunic, which has always been described as a thin, smooth, polished, and transparent membrane—a description which is true, indeed, in the case of the pulmonary artery, but not of the aorta and of its large branches. If we carefully dissect one of these last-named vessels from within, we find, first of all, a thin, transparent pellicle, which lines the vessel—then, exterior to this, is a tissue, hard, dense, and brittle, and which can be raised only in scales or laminæ; M. Margaine has called it the "tunique sclereuse," and described it as a distinct tunic, composed of several foliola, or layers, which are situated between the internal serous, and the elastic or fibrous coats. Now, it is the presence of this coat in the systemic arteries which gives them a greater consistence than the pulmonary arteries, and which causes them to gape, when divided, so much more than these last-named. It is this coat in which the usual morbid changes of arterial tissue, such as the deposition of chalky, steatomatous, cartilaginous, and osseous matter, take place primarily, and the immunity of the pulmonary arterial system from these diseases, is attributable solely to the absence of this peculiar "nidus." When an artery becomes affected with aneurism, the different tunics are more or less changed in their texture and properties: generally they are thickened, softened, and "parsemées" with concretions; the elasticity of the vessel is gone, and, from being unable to resist the extending force of the current of the blood, it becomes gradually dilated, either on one side only, or in all its contour. It is admitted now by almost all pathologists, that sometimes, but not often, the three, or rather, we should say, the four tunics are cotemporaneously dilated. More frequently, however, the internal ones have given way, and the aneurismatic sac consists of the external or cellular coat alone. Without further comment, we now proceed to give a short exposition of M. Amussat's ingenious method of arresting arterial hæmorrhage by torsion: Although this method is applicable chiefly to vessels which have been divided, and gape with open mouths, yet, as it happens occasionally that we designedly cut across a vessel in its continuity, in order to prevent the danger of its bursting, the importance of M. Amussat's suggestion becomes still more conspicuous. Some of the archaiologists of our profession have, with their accustomed intoleration of every thing new and modern, attempted to dispute the claims of this distinguished surgeon to the merit of originality, on the ground that there are a few dark and shadowy hints at similar doings among one or two of the all-learned ancients; but we are not disposed to cavil; and shall leave both the ancients and their advocates "alone in their glory."

If we lay hold of an artery in the dead subject with the forceps, and twist it round in the line of its axis, the spiral fibres of the vessel are first stretched and extended; and, if the twisting be carried further, they become lacerated. If we examine, then, the parts attentively, we shall find that the outer or cellular coat forms a sort of sac, or envelope, which exhibits at its extremity a small knob, "tourillon," formed by the torsion of its parietes; that the middle and inner coats are divided irregularly across, above the point where the forceps were applied; that they are drawn together, detached more or less from the outer tunic, and sometimes are rolled round one on the other within the cavity of the vessel. These are the appearances we observe in the dead body, and the same are to be seen, if the experiment be performed upon the living. Immediately after the operation, we may see the plugged end of the vessel raised or projected at every impulse of the column of the blood, and we are surprised at the complete resistance of the untied orifice. The formation of the internal clot is not long delayed; this clot adheres by its base to the point of rupture and separation of the two inner coats, and the strength of its adhesion appears to be proportioned to the extent of this rupture:—The subsequent changes need not be specified, as they are quite the same as those which are well known to happen after the application of a ligature. There has been some dispute as to the change which the twisted extremity of the artery ultimately undergoes; M. Amussat supposes that it mortifies, or is absorbed, and then leaves exposed the two inner membranes, "refoulées," and firmly glued together; whereas Schrader thinks it more probable that it becomes converted into fibrous tissue.

The operation of torsion of the smaller arteries is very simple: all that we have to do is to lay hold of the bleeding extremity, and twist it several times round its axis; but it is rather more complicated on the larger. M. Amussat recommends that the surgeon be provided with the following instruments—two common forceps, a forceps "à baguettes" (the branches or legs of which terminate in cylindrical rods, which are to be made very smooth, several lines long, and about a line in thickness), and a forceps "à torsion" (the peculiarity of which is, its being provided with a slide on one of the legs, which moves up and down in a groove on the opposite one, and by which it is opened and closed.) With one of the common forceps we lay hold of the vessel—with the other we detach it from its surrounding connexions, so as to make it project free and unattached for a space of six lines or half an inch; we then exchange this second forceps for the forceps "à torsion," with which the artery is seized transversely, and, while we hold this in our right hand, we seize the vessel, at its highest point of detachment, with the forceps "à baguettes" in our left, and by gentle pressure with its blades, we divide the internal and middle coats; then we are to give a slow rotatory movement to the forceps "à torsion," so as to effect the necessary amount of twisting; when this is properly done, we then either gently push back with the forceps "à torsion" the extremity of the artery into the flesh, or cut off the projecting knob, or "tourillon."

The security of this operation depends greatly upon the proper execution of its "deux derniers temps," viz. of the prehension of the vessel with the forceps "à baguettes," and of the subsequent torsion with the forceps "à torsion." If the two inner coats are duly divided across, they retract considerably, so that the forceps has then hold only of the outer or cellular tunic; the retraction of the inner coats causes the puckering up, or "refoulement," which the French authors allude to, and which is increased at each twist that is made of the outer coat. If we press between our fingers the extremity of the artery behind the point of prehension of the forceps "à baguettes," while we keep twisting it, "on sent comme un coin qui chemine et les écarte." When the operation has been properly performed, we observe the end of the vessel exhibiting a knob, or "renflement," rounded "en forme de calotte," and terminating at the middle in a point, which is like a piece of catgut. If we have an opportunity of examining the interior of the vessel at this stage of the process, we find that the inner coats have formed quite an inverted tube, just in the same way as when we have turned the finger of a glove within itself; this tube, being directed against the stream of the blood, would be rolled back by its impulse, were it not for the torsion of the cellular coat, which enables it to afford "une barrière insurmontable."

M. Amussat has made several experiments, to ascertain the power of resistance which a twisted artery offers to a stream directed from within against it; and he has found that, however strongly the injection was forced in with a syringe, the vessel became merely lengthened and distended, and never gave way so as to permit the escape of the fluid. It is to be observed that this perfect resistance occurred in those cases only, in which the "refoulement" of the inner coats had been duly effected, and that, when the torsion merely had been made, without the necessary "refoulement," the injected fluid did sometimes force its way, and become effused in the cellular coat, without, however, having untwisted it.

Some surgeons have thought M. Amussat's directions for performing the torsion of the large arteries too minute and complicated, and, regarding it unnecessary to keep the vessel fixed with a "forceps à baguettes," they dispense altogether with its use; they content themselves with merely laying hold of and drawing out the bleeding extremity with a common forceps, and then, with another (having previously committed the first to the left hand), detaching its connexions and twisting it, till the inner coats are felt to give way—eight or nine turns are generally sufficient, "car il n'est plus douteux qu'alors la valvule externe (il appelle ainsi le cul-de-sac que forme la celluleuse) ne soit formée de tours de spirales suffisans, qui la rendent propre à resister à l'impulsion du sang." If we do not make a sufficient number of turns or rounds in twisting a large artery, there is considerable risk of hæmorrhage, for "les spirales," being neither sufficiently strong nor numerous, may become undone or untwisted (se defont) in consequence of the impulsions of the stream of blood. In the case of the smaller arteries, five or six turns with the forceps are generally sufficient. Before we leave the subject of torsion of the arteries, we may allude to one of the most absurd of all surgical nouveautés: M. Thierry may describe his own dreamings on a particular sort of torsion, applicable only to undivided vessels in their continuity.—"Dans ce procédé, je souleve le vaisseau avec une aiguille des Deschamps, et je m'en sers, comme d'un tourniquet, faisant executer autant de mouvements de torsion, toujours dans le même sens, que l'exige le calibre de l'artère."

M. T. alludes to his operation having been practised by other surgeons; if it has been, there are surely no records, that we know of, of the attempts. The modification of the operation of torsion which M. Amussat himself has suggested, for the continuity of an artery, is much more ingenious and philosphical. Our readers already understand what the French writers mean by the "refoulement" of the inner coats, which is believed to take place in the second step of the operation of torsion, when the open vessel is seized with the forceps "à baguettes;" we shall now briefly explain how M. A. recommends its adoption in the case of an undivided artery:—The surgeon must have two forceps "à baguettes;" he first introduces the blade of one under the exposed and bared artery from one, say the right side, and then introduces a blade of the second from the other, or left side, so that the handles are opposed to each other; the operator holding a forceps in each hand, and bringing the two subjacent blades close to each other, closes both forceps tightly, so as to squeeze the vessel between their blades, for the purpose of rupturing its inner coats; he then, while keeping the upper (nearest the heart) forceps firmly in its place, moves the blades of the lower one obliquely up and down, so as to detach the inner coats from the outer one, and to "refouler les membranes rompues en se dirigeant vers les capillaires." The inversion of the inner coats is to be made always on the side most removed from the heart. In performing this operation of "refoulement," it is necessary that the blades of both forceps should be extremely smooth, for the slightest abrasion of the outer coat might facilitate its rupture, and thus occasion hæmorrhage.

If we examine an artery immediately after the "refoulement" has been performed on it, we find that, at the point corresponding to the point d'appui of the upper forceps, a complete division of the inner coats, and a narrowing of the tube, which is formed now only by the outer or cellular coat; tracing the vessel downwards, we observe the inverted and reverted inner coats, like an irregular cylinder or cone, pointing towards the capillaries, and "du coté du cœur est sa base, espece de petite bourrelet arrondi, que forment les tuniques en se repliant sur elles-memes: tout autour est une petite depression circulaire, point d'union avec la celluleuse, au centre l'entrée du petit tube interne un peu evasée."

M. Amussat is of opinion that a stream of blood continues to pass along the artery, through the "petit canal central des membranes refoulées," for one or two days after the operation, but that, at the end of the second day, the coagulum is formed, and quite obstructs the current; that, by the fourth day, the coagulum has become complete, and adheres powerfully along the whole extent of the denuded cellular tunic, forming a cone which points away from the heart. The exterior of the artery, where it has been detached from its surrounding connexions, generally exhibits traces of suppuration. When the cure is completed, a "fusiforme renflement" remains at this place; on no occasion has the denuded portion of the cellular coat become dilated or aneurismatic, as we might, à priori, be afraid of—indeed M. Amussat has frequently attempted to induce this diseased state in animals, but has never yet succeeded. The operation of complete "refoulement" has almost uniformly been followed by the obliteration of the canal, in the lower animals; but hitherto a trial of it has not been fairly made on the human subject—the ingenious contriver attempted it once, but, being somewhat afraid of employing the requisite force in the manœuvres upon the artery, the "refoulement" of the inner coats was incomplete, and the tube was not, therefore, obliterated.

Although, probably, it may seldom or never be performed, the details of

Amussat's investigations cannot fail to be interesting to the practical as well as to the physiological surgeon. There is still another result of these investigations which remains to be mentioned. We have already explained that an exposed artery, when squeezed between the rounded blades of a forceps "à baguette," has its inner coats divided, while the outer one remains unbroken. Now, if we do nothing more than merely this manœuvre, and leave the vessel in this state, each wave of the blood as it passes along washes the jagged edges of the wounded tunics, and thus preventing the deposition and adhesion of lymph, a coagulum is not formed. M. Amussat has never succéeded in procuring the obliteration of an artery, however many of these "machures," (we are almost obliged to coin a new word—shall we call it "morsion," as opposed to "torsion?") and in whatever direction they be made. On examining the interior of the vessel, a short time after the operation, he found a number of small cicatrised wounds, which gave the appearance of unevenness and irregularity to its surface.

Similar phenomena are observed after the temporary application of a ligature; and indeed this has all the effects of the "machure" now alluded to. In either case no coagulum is formed, and the results therefore of the operations are imperfect and unsatisfactory. M. Amussat, during his inquiries on this subject, having ascertained that such was the progress of things under these circumstances, was then anxious to determine what changes would take place, if the current of blood along the affected artery was arrested for some time after the "machures" had been made; and for this purpose he put a ligature on the artery below, or on the side of the capillaries.

The carotid artery of a large dog being exposed, he made two divisions or "machures" of the inner coats, two lines apart from each other, and then applied a ligature, two or three lines distant from the lower or distal "machure."

The usual conical coagulum was formed in this case, with its base adhering to the site of the ligature, and its apex towards the heart; but the peculiarity was, that at each "machure" the coagulum adhered by a circular "embranchement" to the edges of the divided membranes. There were therefore here three firm and solid adhesions of the coagulum, and the resistance therefore to the impulsion of the blood was threefold strong. At the spaces intermediate between the different "machures," the coagulum was perfectly free, and so unattached, that a probe might be passed between it and the inner surface of the artery. The truth of this description M. Amussat has verified by numerous experiments. In every case, without exception, has it been found, that the coagulum had formed a distinct and separate adhesion to each "machure." At the end of four days, as the adhesions then are generally firm and well organised, the ligature may with perfect safety be removed, and the wound be then healed as quickly as possible. As yet, no trial of the operation in question has been made on the human subject; but the results on animals have been so uniform, and so satisfactory, that we may fairly anticipate some important advantages from its adoption in certain cases, and perhaps more especially in those in which there is reason to apprehend secondary hæmorrhage, in consequence of some collateral branch being given off by a tied artery, near the site of the ligature. Such is an abstract of M. Lisfranc's observations on the means of arresting hæmorrhage from an open artery, and of plugging up one in its continuity by the operations of "torsion" and "refoulement." Hitherto the subject has scarcely received in this country the attention which it deserves; and we have been therefore induced to extend our review to a greater length than we at first proposed. The rest of M. Lisfranc's Essay can be merely glanced at. The relative frequency of aneurism in the different arterial trunks, and at different periods of life, is shewn in the following Tables. In 179 cases—

| | | | |
|---|---|---|---|
| Artery—popliteal | 59 | Artery, Humeral | 3 |
| ——— Crural, at the bend of the groin | 26 | ——— Common iliac | 3 |
| ——— Crural, at different points, above and below the groin | 18 | ——— Ant. tibial | 3 |
| ——— Carotid | 17 | ——— Gluteal | 2 |
| ——— Subclavian | 16 | ——— Int. iliac | 2 |
| ——— Axillary | 14 | ——— Temporal | 2 |
| ——— External iliac | 5 | ——— Int. carotid | 1 |
| ——— Brachio-cephalic | 4 | ——— Ulnar | 1 |
| | | ——— Fibular | 1 |
| | | ——— Radial | 1 |
| | | ——— Palmar | 1 |

In 101 cases, in which the ages of the patients are faithfully recorded, we find—

| | | | |
|---|---|---|---|
| 1 case, at | 13 years. | 20 cases, at | 40—45 years. |
| 3 | 15—20 | 17 | 45—50 |
| 5 | 20—25 | 11 | 50—55 |
| 12 | 25—30 | 6 | 55—60 |
| 24 | 30—35 | 3 | 60—70 |
| 15 | 35—40 | 3 | 70—80 |

An ingenious but fanciful hæmostatic remedy has been proposed by M. Pravas: it is the employment of galvanism and acupuncture together. Since the experiments of Scudamore, it has been very generally known that blood, when submitted to the electrical current, very speedily coagulates; and M. M. Pravas and Guerard were induced to examine the subject more minutely. They have ascertained that, if even the aorta of a rabbit be wounded, the hæmorrhage may, at least for a time, be arrested by the agency of galvanism. "No sooner were the wires applied to the bleeding vessel than a brown-coloured clot was formed."

"Is a trial," says our author, "of this innocuous means not deserving to be made on an aneurismatic swelling?" Nothing could be more easy, than the introduction of one or more acupuncture needles, and the establishing a current of galvanism through the contents of the sac. The hint is at all events worthy of notice, and may possibly, in some cases, be turned to a useful account.

Even acupuncture alone has been proved by M. Velpeau to have considerable power in inducing coagulation of the blood in the living subject. He introduced a needle into the crural artery of a dog, and found on the fifth day that a firm fibrinous concretion plugged it up completely for the extent of one inch, or thereabouts. This experiment was repeated several times, and the invariable result was, that when the needle or needles were retained in an artery for four days at least a solid coagulum was formed, and an obliteration more or less complete was effected. It is to be remembered that M. Velpeau never experimented on any vessel larger than the femoral of a middle-sized dog.

The subsequent trials made by M. Amussat on the carotid arteries of horses have been less favorable to the success of acupuncturation. He passed four long needles through the carotid of a horse, in different directions, and allowed them to remain in situ for sixty hours. The animal was then killed, and M. A. discovered traces of considerable inflammation around the vessel, on opening which, all the needles were observed to be quite smooth and shining, but there was no appearance of coagulation having ever taken place.

Besides this negative objection, there is the danger of hæmorrhage being induced by wounds of the artery, however small these wounds may be. Guthrie alludes to a case where the carotid artery had been wounded by a needle and required a ligature, and to two others, in which the femoral artery, having been wounded by a tenaculum, became the seat of ulceration, and hæmorrhage ensued.

Appended to M. Lisfranc's memoir is a very extended and most valuable analytical table of upwards of 260 cases of aneurism and of wounded arteries, which shews at one view the name and age of each patient, the artery affected, the operation or other treatment followed, the occurrence of secondary hæmorrhage, or of other accidents; the date of the falling off of the ligature, the ultimate issue, and, lastly, the bibliographical source, whence the original report of each case may be derived.

Well may our indefatigable author assert "that this table is an authentic clinical commentary on most of the opinions and reasonings contained in his Essay, and will be a useful pattern for young surgeons to imitate in their study of surgical science. Facts are worth every thing else. Every conclusion which is not drawn from facts must be wavering and uncertain."

Five cases of aneurism, treated on Valsalva's plan, are first on the list; then twelve cases treated with refrigerants and styptics; then forty-seven, in which compression, after various methods, had been employed; thirty, in which the old operation of opening the aneurismatic sac, &c. had been performed; 180, treated by Anel's (or, as we call it in this country, Hunter's) operation; and lastly, fourteen, in which Brasdor's operation was adopted. The comparison of the results of these different modes of treatment is very instructive. Out of the 180 cases, in which the ligature had been applied according to Anel's or Hunter's method, we observe that secondary hæmorrrhage occurred in 32, which gives the proportion of about 1 in 6. The hæmorrhage took place usually from the sixth to the twenty-fourth day;—in a few cases it was much later, not till the 50th or 60th day after the operation. The number of deaths in the above catalogue of 180 cases amounts to 43; the mortality, as we might, à priori, expect, being much influenced by the size and other relative peculiarities of the vessels tied. The results of Brasdor's operation, as it is called, are well known to have been very generally unfavorable. In fourteen cases referred to, only three patients were saved; and there is doubt as to the nature of the existent disease in one of these.

For the particulars we must refer our readers to M. Lisfranc's table, which we have already praised. It deserves to be consulted by all future writers on aneurism: and indeed the memoir altogether is worthy of the established reputation of the author.

